# The modular organization of roe deer (*Capreolus capreolus*) body during ontogeny: the effects of sex and habitat

**DOI:** 10.1186/s12983-018-0283-8

**Published:** 2018-09-27

**Authors:** Svetlana Milošević-Zlatanović, Tanja Vukov, Srđan Stamenković, Marija Jovanović, Nataša Tomašević Kolarov

**Affiliations:** 1 Faculty of Science, University of Kragujevac, Radoja Domanovića 12, Kragujevac, 34000 Serbia; 2 Department of Evolutionary Biology, University of Belgrade, Institute for Biological Research “Siniša Stanković”, Bulevar despota Stefana 142, Belgrade, Serbia; 3 Faculty of Biology, University of Belgrade, Studentski trg 16, Belgrade, Serbia

**Keywords:** Modularity, Morphological covariation, Morphological integration, Evolvability

## Abstract

**Background:**

As a small artiodactyl, the roe deer (*Capreolus capreolus* L.) is characterized by biological plasticity and great adaptability demonstrated by their survival under a wide variety of environmental conditions. In order to depict patterns of phenotypic variation of roe deer body this study aims to quantify variation during ontogenetic development and determine how sex-specific reproductive investment and non-uniform habitat differences relate to phenotypic variation and do these differential investments mold the patterns of phenotypic variation through modular organisation.

**Results:**

Patterns of phenotypic correlation among body traits change during the ontogeny of roe deer, with differential influence of sex and habitat type. Modularity was found to be a feature of closed habitats with trunk+forelimbs+hindlimbs as the best supported integration/modularity hypothesis for both sexes. The indices of integration and evolvability vary with habitat type, age and sex where increased integration is followed by decreased evolvability.

**Conclusion:**

This is the first study that quantifies patterns of correlation in the roe deer body and finds pronounced changes in correlation structure during ontogeny affected by sex and habitat type. The correlation structure of the roe deer body is developmentally written over the course of ontogeny but we do not exclude the influence of function on ontogenetic changes. Modularity arises with the onset of reproduction (subadults not being modular) and is differentially expressed in males and females from different habitats. Both adult males and females show modularity in primordial, closed habitats. Overall, all these findings are important as they provide support to the idea that modularity can evolve at the population level and change fast within a species.

## Background

Organismal form is one of the complex morphological structures that include not just the shape of anatomical parts, but also their size, arrangement, relative orientation, and connections of these parts [1]. Understanding how complex morphological structures arise during development and how they are altered during evolution is not a simple task. One of the important breakthroughs in understanding of the evolution of organismal form is discovering phenotypic modules [2, 3]. A module represent a part of an organism that is integrated with respect to a certain kind of process (natural variation, function, development) and relatively autonomous with respect to other parts of the organism [4–6]. Modularity allows evolutionary change within modules without profound altering function or structure of other modules [5, 7]. It enhances the capacity to generate morphological variation by overcoming internal constrains such as physical limits imposed by biomechanics on organismal size and shape and developmental constraints that limit the range of variation. Therefore, depicting modular organization is crucial for better understanding of morphological diversity, phenotypic evolution, and evolvability [8, 9].

The animal body is a complex structure comprised from different genetic, developmental, functional and evolutionary modules constructed on a body plan. Modularity of the body as a whole was out of focus in contemporary eco-evo-devo studies, although a large number of studies of the modularity of different body parts exists, on both, inter- and intraspecific levels [10–18].

Roe deer (*Capreolus capreolus* L.) is very interesting species for eco-evo-devo studies as it is widespread ungulate species in Europe with high level of flexibility and success in colonizing different habitats. Adaptation to wide variety of environments and habitats influenced the social organization and spatial behaviour of roe deer populations [19], where availability and configuration of woodland habitats have an important role. Differences in social and spatial behaviour of roe deer populations in open and closed habitats have led to a long-standing distinction in between “forest” and “field” roe deer based on morphological and genetic variation [18, 20–24].

The body of roe deer has been studied in light of relationships between body size/mass and life history traits [25, 26], environmental variation [27, 28], habitat characteristics [27], and population parameters [29–31]. Similar to most vertebrates, populations of a roe deer are strongly age structured [32] and therefore natural selection optimizes the most efficient combination of body traits allowed by developmental constraints and environmental filters acting at each ontogenetic stage. Most studies of roe deer morphological variability throughout ontogeny deal with body size. Fast juvenile growth is followed by sex-specific resource allocation related to different reproductive schedules [33]. The timing of the first reproductive investment constitutes a major physiological and energetic constraint which has a large impact on the ontogeny of sex differences in body size and mass. In addition, the growth and resource allocation strategy in roe deer is highly susceptible to habitat quality [33–35]. Poor habitat quality results in decreased body sexual size dimorphism [33], where the younger age classes are usually more sensitive to environmental variation because of their requirements for rapid growth [36, 37]. However, little attention has been paid to the question of how does the roe deer body as a complex morphological structure emerge during ontogeny through the prism of modularity.

In this study we explored modularity and morphological integration of the roe deer body traits across two ontogenetic stages (subadults and adults). We expect more pronounced body modules in sexually mature adults as resources are differentially (sex-specific) allocated from intense growth and channelled into improving functional performances. As this species is well adapted to wide variety of environments and habitats with complex social organization and spatial behaviour correlated to sex [19], we also addressed impact of habitat and sex on the roe deer body modular organization during ontogeny. We think that in this species, which is known to be phenotypically plastic, it is important to ask how phenotypic variation is organised: is it uniform during ontogenetic development, how sex-specific reproductive investment and non-uniform habitat differences relate to phenotypic variation and do these differential investments mold the patterns of phenotypic variation through modular organisation.

## Results

### Multivariate morphological differences

**Fig. 1:**
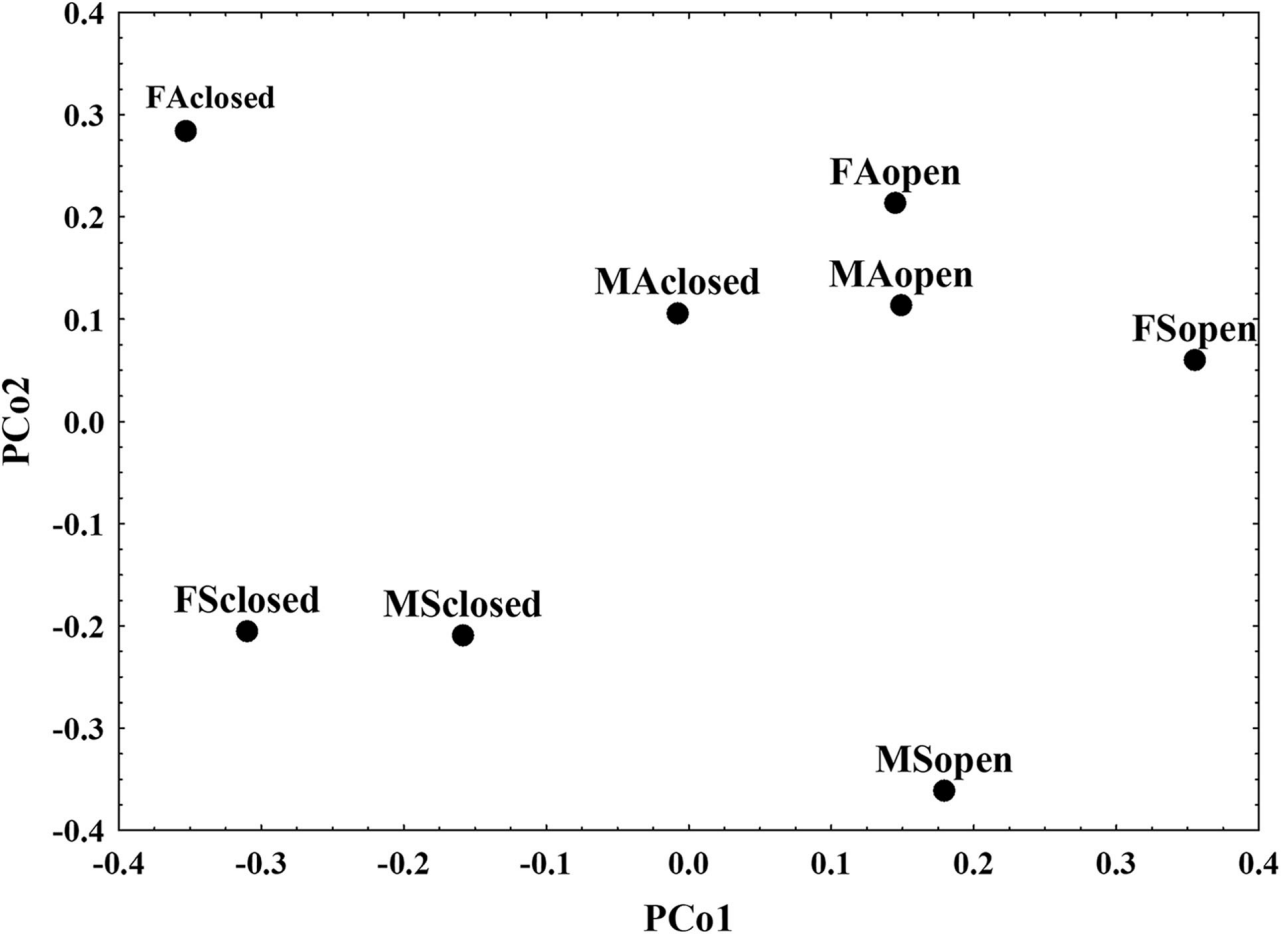
Graphical presentation of first two axes from a Principal coordinate analysis (PCoA) of distances between eight sex/age/habitat group matrices

A three-way MANOVA revealed significant main effects of habitat (Wilks’ λ = 0.2984; d.f. = 14, 549; *P* < 0.0001), age (Wilks’ λ = 0.7023; d.f. = 14, 549; *P* < 0.0001), and sex (Wilks’ λ = 0.4423; d.f. = 14, 549; *P* < 0.0001) and significant interaction effects for all two-way interactions as well as the three-way interaction (*P* < 0.0001). A principal coordinate analysis for the distance matrices of the eight identified groups was performed to further explore the pattern of multivariate differentiation (Fig. 1).

As readily observed from the Fig. 1, the first two PCoA axes show that the eight groups differentiate as follows: the first PCoA axis separates habitat groups, with groups from open habitats having positive while groups from closed habitats having negative scores; the second PCoA axis separates age groups with adults scoring positively while groups with subadults scoring negatively (except FSopen). The absence of clearly defined clusters, suggests complex patterns of differentiation and is corroborated by the significance of interactions in the three-way MANOVA. Therefore, further analyses have been done on eight sex/age/habitat groups: Female/Subadult/Open (FSopen), Female/Subadult/Closed (FSclosed), Female/Adult/Open (FAopen), Female/Adult/Closed (FAclosed), Male/Subadult/Open (MSopen), Male/Subadult/Closed (MSclosed), Male/Adult/Open (MAopen), Male/Adult/Closed (MAclosed).

### Patterns of correlation

To assess correlation patterns in eight sex/age/habitat groups, Pearson correlation matrices were generated for each group using 14 allometricaly adjusted body traits.

Correlation matrix repeatability was high, with values over 0.90 in all cases indicating a robust data set for each sex/age/habitat group of roe deer (lower values were found in subadult males and females within closed habitats) (Table 1).

**Table 1:** Results of matrix correlation analysis for body traits of sex/age/habitat groups of roe deer

age			Subadult				Adult			
	habitat		open		closed		open		closed	
		sex	females	males	females	males	females	males	females	males
Subadult	open	females	0.95	*0.00*	*0.02*	*0.06*	*0.00*	*0.00*	*0.06*	*0.00*
		males	0.37	0.94	*0.01*	*0.00*	*0.01*	*0.00*	*0.08*	*0.00*
	closed	females	0.24	0.27	0.91	*0.01*	*0.07*	*0.01*	*0.01*	*0.00*
		males	0.18	0.44	0.35	0.90	*0.00*	*0.00*	*0.01*	*0.00*
Adult	open	females	0.38	0.29	0.17	0.41	0.97	*0.00*	*0.00*	*0.00*
		males	0.45	0.37	0.30	0.36	0.51	0.97	*0.00*	*0.00*
	closed	females	0.18	0.16	0.32	0.32	0.33	0.32	0.93	*0.00*
		males	0.49	0.39	0.39	0.40	0.43	0.54	0.48	0.94

Values left to the diagonal are correlations, the diagonal is repeatability and right to the diagonal are *p*-values from Mantel’s test (in italics)

Mantel’s test was used to show whether the group matrices were more similar to each other or to randomly generated matrices. The results revealed complex relations between all factors analyzed in this study with almost all paired comparisons significant (only 4 out of 28 paired comparisons were non-significant). Subadults had lower correlations then adults. The highest correlations in both age groups were between males from open and closed habitats. The same pattern characterized subadult and adult groups: sexes were more similar in open habitats, males from different habitats were with higher correlations in relation to females, and correlations from open habitats were higher than from closed habitats (Table 1).

**Fig. 2:**
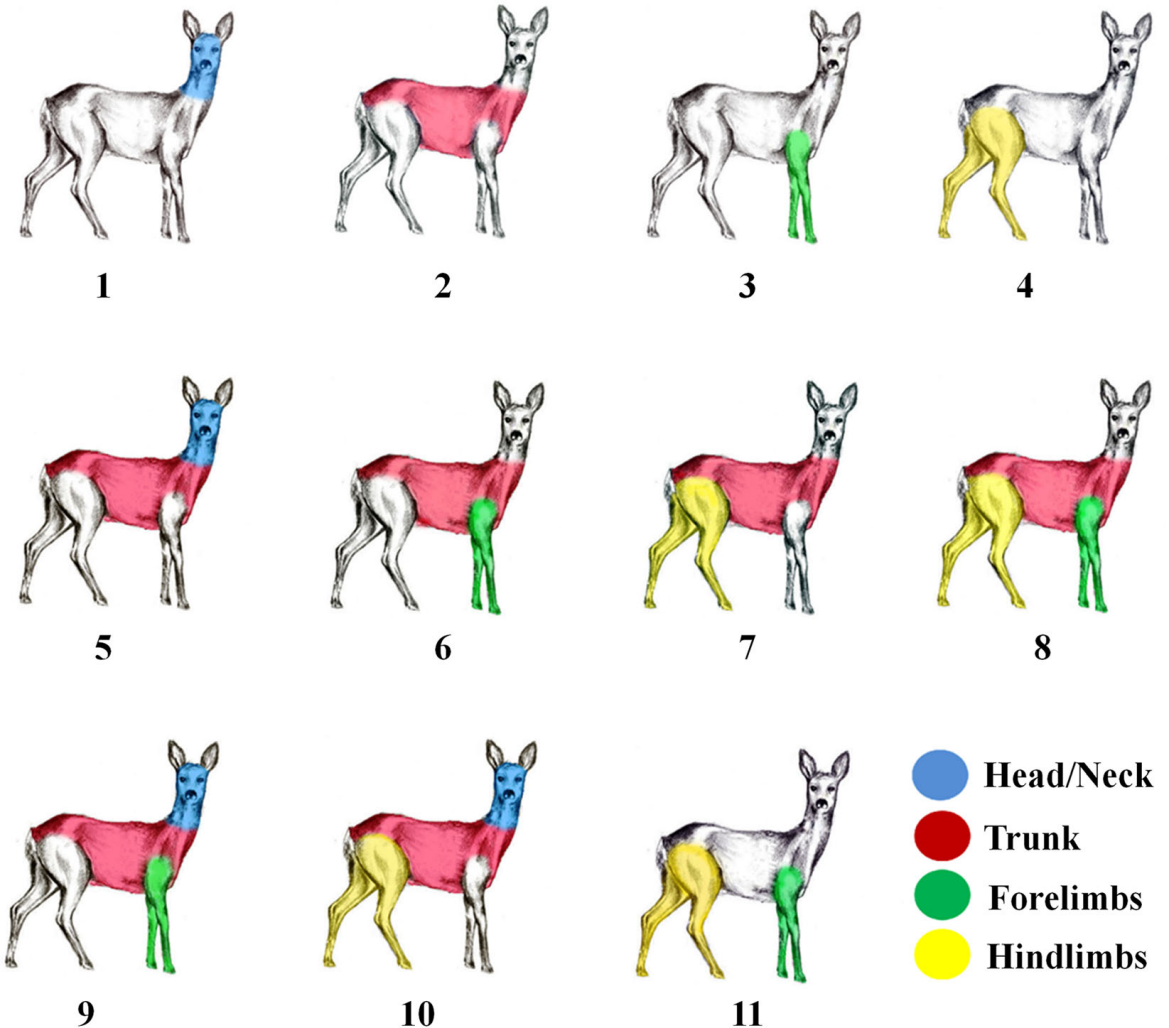
Graphical representation of the 11 a priori hypotheses of modularity. Regions of the body sharing the same coloration pattern form putative variational modules. See Table 2 for precise description of hypotheses

To test integration/modularity hypotheses, the sex/age/habitat group correlation matrices were compared to connectivity matrices representing hypothesized integration of body traits. Out of the eleven hypotheses (Fig. 2), only five were statistically significant and present in adult males and females from closed habitats. Correlations of trunk+forelimbs+hindlimbs (FAclosed: *r* = 0.32; *p* = 0.004; MAclosed: *r* = 0.22; *p* = 0.017) and correlations of forelimbs+hindlimbs (FAclosed: *r* = 0.29; *p* = 0.001; MAclosed: *r* = 0.21; *p* = 0.022) were significant in adult females and males from closed habitats. Additionally, we observed three significant correlations of modules in adult females from closed habitats: trunk+hindlimbs (*r* = 0.23; *p* = 0.014), forelimbs (*r* = 0.22; *p* = 0.030) and trunk (marginally significant *r* = 0.17; *p* = 0.080). Taking into account only these significant integration/modularity hypotheses, we compared their maximum likelihoods in order to determine which of the hypothesis better explains the data. The preferred hypothesis was trunk+forelimbs+hindlimbs in both adult females (ML = 56.6; AICc = − 106.9; posterior probability = 0.72) and males (ML = 8; AICc = − 9.66; posterior probability = 0.52) from closed habitats. Correlations of head/neck+trunk were of marginal statistical significance in adult females and males from open habitats (FAopen: *r* = 0.11; *p* = 0.083; MAopen: *r* = 0.17; *p* = 0.066).

### Partial correlations

**Fig. 3:**
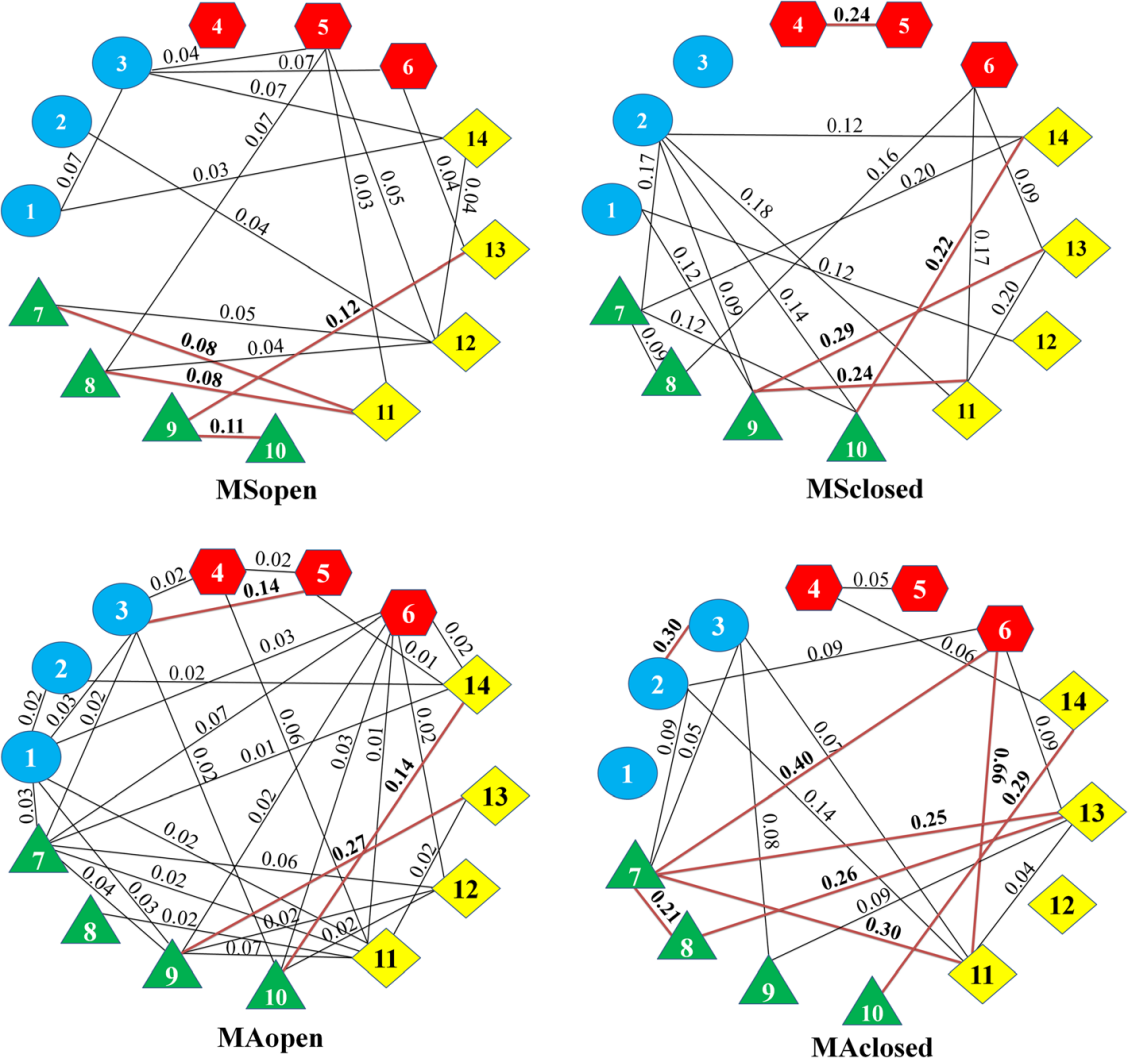
Conditional independence graph for a basic module organization of male roe deer body within age/habitat groups. Only significant edges are illustrated. The numbers in bold above red line indicate particullarly strong edges. The numbers in geometric shapes refer to the body characters included in each partition according to Fig. 7. Blue circles indicate head/neck module, red hexagons indicate trunk module, green triangles indicate forelimbs module, yellow squares indicate hindlimbs module

**Fig. 4:**
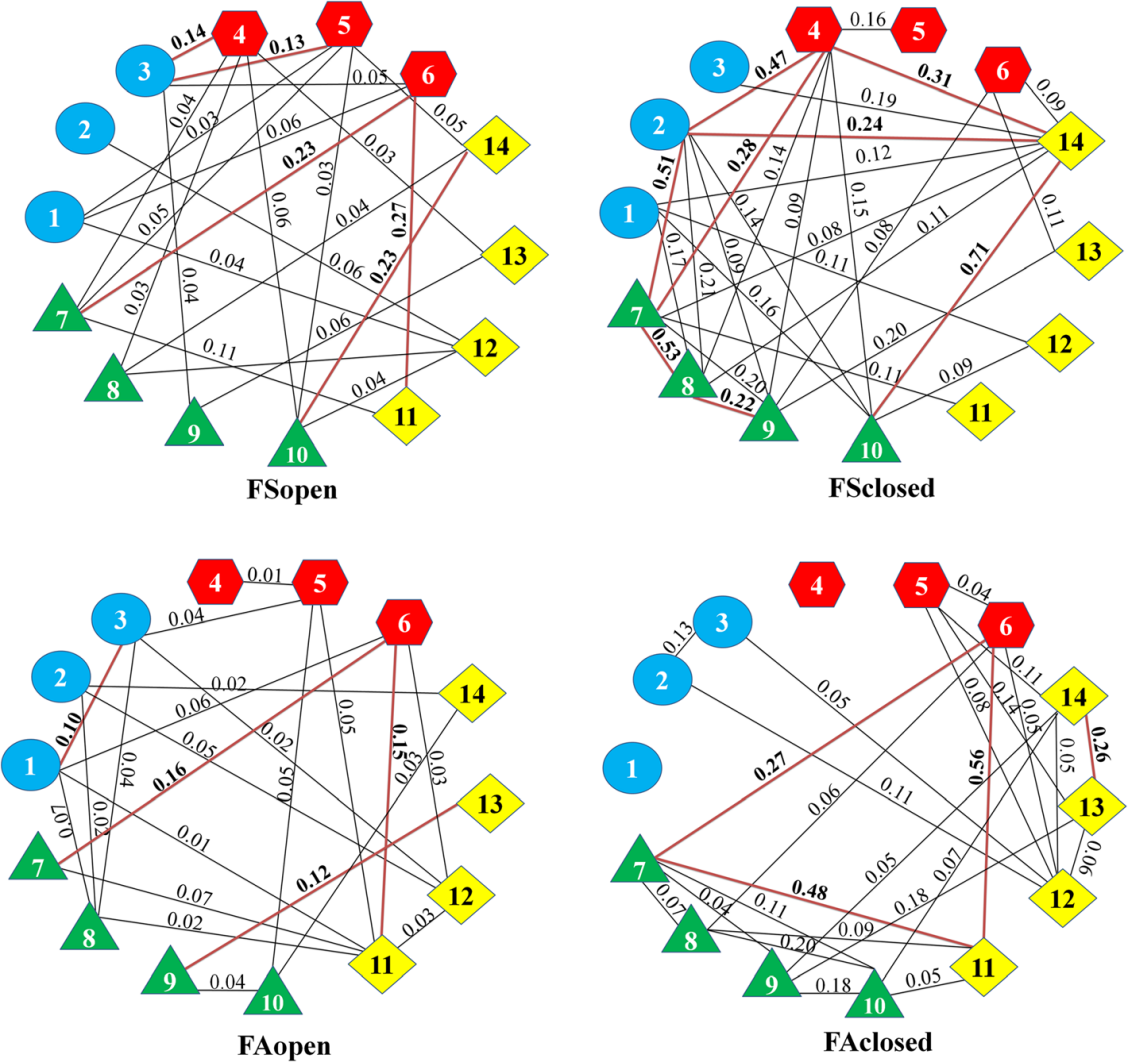
Conditional independence graph for a basic module organization of female roe deer body within age/habitat groups. Only significant edges are illustrated. The numbers in bold above red line indicate particullarly strong edges. The numbers in geometric shapes refer to the body characters included in each partition according to Fig. 7. Blue circles indicate head/neck module, red hexagons indicate trunk module, green triangles indicate forelimbs module, yellow squares indicate hindlimbs module

Partial correlations measure associations between two traits that remain when underlying, shared correlations with other traits have been removed. To visualize the results of partial correlation analysis, conditional independence graphs were constructed. Conditional independence graphs for subadult and adult males and females from different habitats are shown in Figs. 3 and 4. All illustrated edges in these graphs are significant with bold lines highlighting strong edges. Less than 35% of all potential edges were present. These graphs revealed several patterns common for all groups. The highest partial correlations or strong edges tend to be within trunk and limbs or between fore- and hindlimbs, specifically they include the links between “chest depth” and “forelimb length” or “hindlimb length”, and between “hoop length of left forelimb” and “hoop length of left hindlimb”.

The average connectivity per trait was not significant between sex, age or habitat groups, but there was a significant age*sex effect (F = 5.3; *p* = 0.022). This indicates an absence of parallelism of the ontogenetic integration between males and females. In males, average connectivity increases from subadults to adults. The pattern is opposite for females, in which the highest average connectivity was found for subadults.

The edge strength was statistically significantly different between subadults and adults (F = 6.04; *p* = 0.016) and between habitats (F = 63.2; *p* < 0.001). Subadults and individuals from closed habitats had higher edge strength than adults and individuals from open habitats.

### Index of integration

**Fig. 5:**
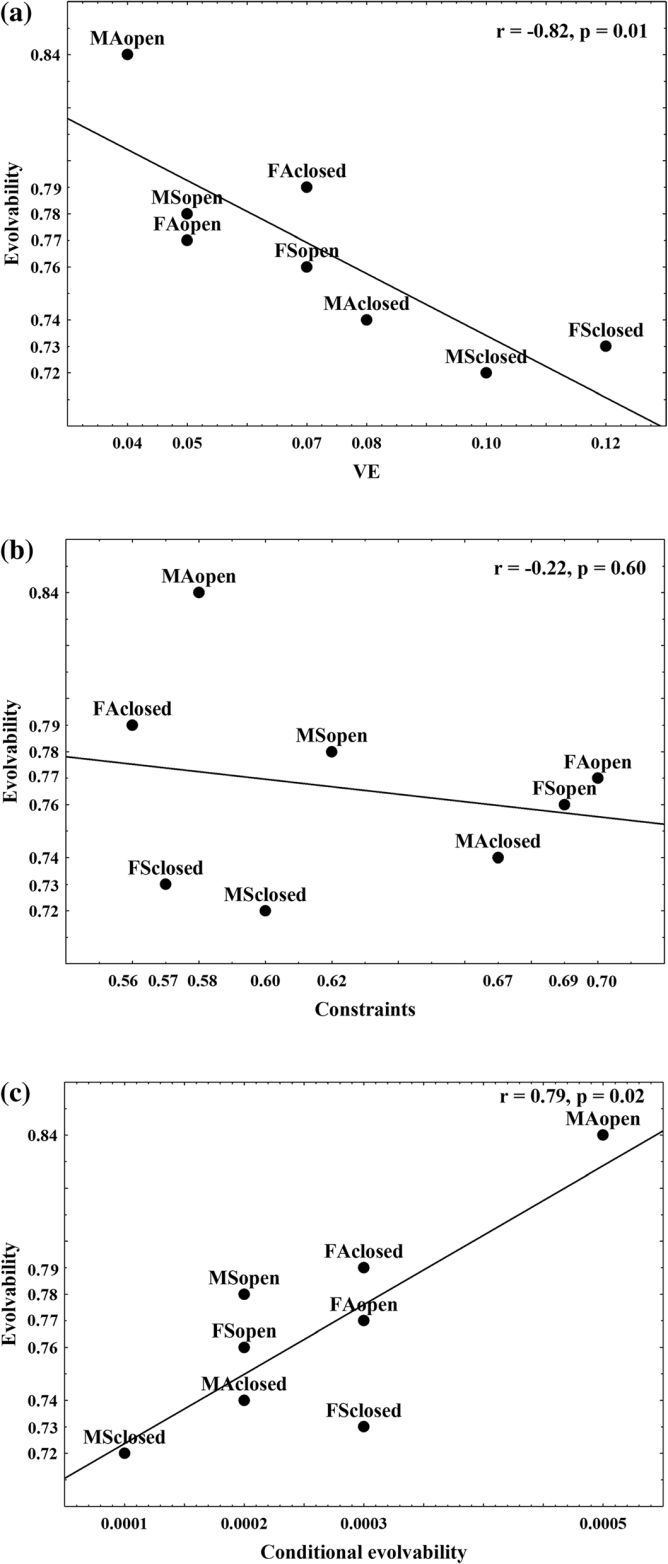
**a** Plot of evolvability against index of integration (VE), **b** plot of evolvability against constraints, **c** plot of evolvability against conditional evolvability. The values on x and y axis relate to group values for index of integration (VE), evolvability, conditional evolvability and constraints

The standardized variance of the eigenvalues (VE) was used to assess overall covariation of body parts. Our results indicated variable levels of integration between age, sex and habitat groups. The index of integration ranged from 0.04 to 0.12 (Fig. 5). Subadults had higher VE values in relation to adults. Furthermore, females and individuals from closed habitats generally demonstrate higher VE values in relation to males and open habitats. Pairwise comparisons of VE among groups show statistically significant differences in all cases (*p* < 0.05) (except for the FSopen/FAclosed comparison).

### Evolvability indices

Evolvability indices for each of eight sex/age/habitat groups were evaluated to examine the ability of the body to respond to selection. The mean cosine of the angle between the selection and response vector ranged from 0.72 to 0.84, conditional evolvability ranged from 0.0001 to 0.0005, and constraints ranged from 0.56 to 0.70 (Fig. 5a, b, c). In general, lower VE values were followed by higher evolvabilities (*r* = − 0.82; *p* = 0.01). Thus, adult individuals, males and open habitats showed higher evolvabilities than subadults, females and closed habitats, respectively (except for adults from closed habitats where females had higher values). Pairwise comparisons indicate differences in the ability of body form to respond to selection (statistically significant in all comparisons except between FSopen/FAclosed), regardless of a relatively high level of evolvability in roe deer. The significant relation was found for correlation of evolvability and conditional evolvability (*r* = 0.79; *p* = 0.02), while relation between evolvability and constraints was not confirmed (*r* = − 0.22; *p* = 0.60) (Fig. 5a, b, c).

## Discussion

Ontogenetic dynamics of modularity and integration is very important in eco-devo studies as they have significant consequences for morphological variation. However, a relatively small number of studies have been focused on this aspect [38]. Most of the studies confirm repatterning in modularity during ontogeny followed by modularity increase during development [39–42]. Others find no significant changes in variance or integration during ontogeny [43, 44]. Changes in modularity and integration during ontogeny could exist, on the basis of predominant theoretical considerations, (1) if morphological integration matches functional integration and therefore morphological integration should change along with function [45] or (2) if developmental processes in each successive stage produce different correlation structures and therefore correlation patterns at any ontogenetic stage are a function of cumulative effects over all previous stages - palimpsest model [46]. On the other hand, (3) development canalized to meet the functional demands of the adult could explain lack of variation in correlation structure during ontogeny.

Our results suggest that patterns of phenotypic correlation among body traits change during the ontogeny of roe deer, with modular structures emerging in adults. In our opinion, this basic finding corroborates Hallgrímsson’s palimpsest model where an integrated phenotype is developmentally written over the course of ontogeny [46] (consideration 2), although we acknowledge that consideration (1) could set the stage for the main differences in patterns of modularity during ontogeny, where the shift in correlation structure is related to the onset of reproduction. In roe deer, like in all cervids, sexual differences result from differing reproductive strategies, which include differential predation risks, activity budgets and social organization [47]. Individuals change their “focus” from rapid growth to reproduction where reproductive success of males depends on their physical condition with a consequence that they select higher quality habitat patches regardless of the risk of predation. The success of females is correlated with the survival of their offspring, which are more vulnerable to predation than the adults, with females selecting habitats with more protective covering. We suggest that these different reproductive strategies between males and females could result in different levels of modularity between sexes which is found in the roe deer. Adult roe deer females have more modular body than males with the trunk+forelimbs+hindlimbs hypothesis best supported for both sexes. These findings are not unexpected as integrated movement of trunk and limbs play a central role in locomotion in tetrapods in general [48]. Although trunk role in locomotion is less apparent, it is a foundation for the production of mechanical work by the limbs and is central to the static and dynamic control of body posture, providing integrated actions of limbs and trunk [49, 50]. Limbs, on the other hand, represent serially homologous structures (for different view, see [51, 52]) considered to be highly integrated due to their shared developmental, functional and/or genetic influences [45, 53].

We showed that apart from the impact of sex on levels of body modularity in the adult roe deer, habitat has an important role in shaping patterns of correlation structure. Interrelation between habitat and phenotypic integration results from correlational selection favouring different adaptive trait combinations in different habitats, morphs or ecotypes [54, 55]. Modularity of roe deer body is related to closed habitats. The possible explanation is that the closed habitats are primordial and more stable in the long-term evolution of the roe deer, thus providing for stronger canalizing selection pressures. Open habitats are secondary and heterogeneous leading to wider spectrum of pressures resulting in differential responses to habitat quality which by itself causes lower modularity. Although body modules are not significant in adult roe deer from open habitats, there are higher correlations among characters of the head/neck+trunk module. That could be related to higher awareness of surroundings (vision, olfactory and auditory perceptions) due to bigger predation risk, greater disturbance level, lower level of cover and more time devoted to orientation and social behavior during spring and summer [56, 57].

Contrary to adults, our results show absence of body modular structure in roe deer subadults. They are characterised by rapid growth which can increase integration if the generated variance is highly structured (if the generated variance is random, a decrease in integration during growth is expected). Nevertheless, the foundation for adult modular structure is present in subadults as strong edges within the trunk and limbs modules or between fore- and hindlimbs modules, which can be seen as construction characters that connect different ontogenetic stages creating a constrained body form framework. The body modules in adults are build up “around” these construction characters enabling differential responses to changes in environmentally-driven selective pressures.

The capacity of a population to adapt to selection (evolvability) can be constrained or facilitated by integration patterns. As Cheverud’s model of integration predicts [58], when phenotypic traits become integrated, their responses to selection become more coordinated, while their ability to respond to selective pressures is reduced. However, the relation between integration and evolvability is not straightforward, as the process of dissociation between traits by removing pleiotropic effects can enhance or diminish evolvability of the system as a whole – a pleiotropic effect is only an absolute constraint if the traits are perfectly correlated [59]. Our study showed that evolvability of roe deer body changes during ontogeny, and it is different between sexes and habitats with the general pattern – higher integration implies lower evolvability. Thus, our results confirm that the evolvability of morphological traits depends on how strongly they covary with other traits, which is in concordance with Rolian’s [60] results on primate hands and feet. It should be noted that we measured evolvability as the capacity to respond to selection in any direction in phenotypic space. Theoretical considerations suggest that evolutionary responses are usually channeled along the path of least evolutionary resistance in a phenotypic space determined by size-related variation [60–62], which mostly influences phenotypic evolution of quantitative traits [63]. Patterns of correlation and evolution of correlation structures which do not depend on size are possibly obscured depending on whether size is considered or not. We obtained, on size-corrected data, that evolvability did not significantly follow the lines of least resistance (seen as the correlation between evolvability and constraints), but rather evolved in the direction of stabilizing selection pressures, i.e. developmental constraints (seen as the correlation between evolvability and conditional evolvability). We stress again that all our analyses were conducted on size-corrected data placing emphasis on correlation patterns related to shape rather than size variation.

Our findings are important as they provide support to the idea that modularity can evolve at the population level and change fast within a species. Modularity is often assumed to be a constrained/stable property that only changes over long geological timescales [16], but some studies showed it can vary between closely related species and populations which indicate that modularity has a relatively simple genetic basis, and therefore may respond rapidly to selection [17, 64–66]. Fast responses of roe deer body modularity to differential habitat/sex selection point out that although body shape has a complex genetic basis, the pattern of modularity itself might have a simpler genetic basis [67]. Some studies show the genetic basis of variation and covariation appear to be highly overlapping, which suggests pleiotropy [68] while others indicate genetic decoupling of phenotypic variation and covariation [69, 70]. Decoupling of body variation and covariation may be a property that allows roe deer populations to avoid tradeoffs that occur under pleiotropy and increase their evolvability in certain habitats. However, since we have no data on the genetic basis of developmental modularity in the roe deer, we cannot discount the influence of purely epigenetic influences due to feeding, locomotion and social behavior (diet, growth, musculature) which are in general incorporated into the concept of phenotypic plasticity. Future research should aim to identify and quantify the genetic basis and developmental structures that constrain and facilitate modularity and evolvability in different ecological contexts.

As a small artiodactyl, the roe deer is characterized by biological plasticity and great adaptability demonstrated by their survival under a wide variety of environmental conditions [18, 71–76]. The body as an integrated unit responds to selective pressures of variable environmental conditions through production of reorganized phenotypes. In the case of the roe deer, body modules emerge with adulthood and body modularity varies across sexes and habitats. These findings indicate that modularity can evolve at the population level and change fast within a species under differential selection pressures. In conclusion, we suggest that the ecological and evolutionary success of the roe deer, especially in the context of post-pleistocene recolonization of Europe, as well as its adaptability to large-scale anthropogenic habitat modifications, is primarily due to a non-uniform and flexible organization of phenotypic variation which enables it to respond quickly to differential sex/habitat contexts.

## Conclusions

In this study we explored modularity and morphological integration of the roe deer body traits across two ontogenetic stages: subadults and adults. As this species is well adapted to wide variety of environments and habitats with complex social organization and spatial behaviour correlated to sex, we also addressed impact of habitat and sex on the roe deer body modular organization during ontogeny. Our results indicate pronounced changes in correlation structure during ontogeny affected by sex and habitat type. We suggest that different reproductive strategies between males and females could result in different levels of modularity. Modular organisation is related to closed habitats as a consequence of a more stable environment in comparison to open habitats, thus providing for stronger canalizing selection pressures. The best supported integration/modularity hypothesis is trunk+forelimbs+hindlimbs for both sexes probably due to importance of integrated movement of trunk and limbs in locomotion and shared development between fore- and hindlimbs. Our results confirm that the evolvability of morphological traits depends on how strongly they covary with other traits. Overall, all these findings are important as they provide support to the idea that modularity can evolve at the population level and change fast within a species.

## Methods

### Sample and data collection

**Fig. 6:**
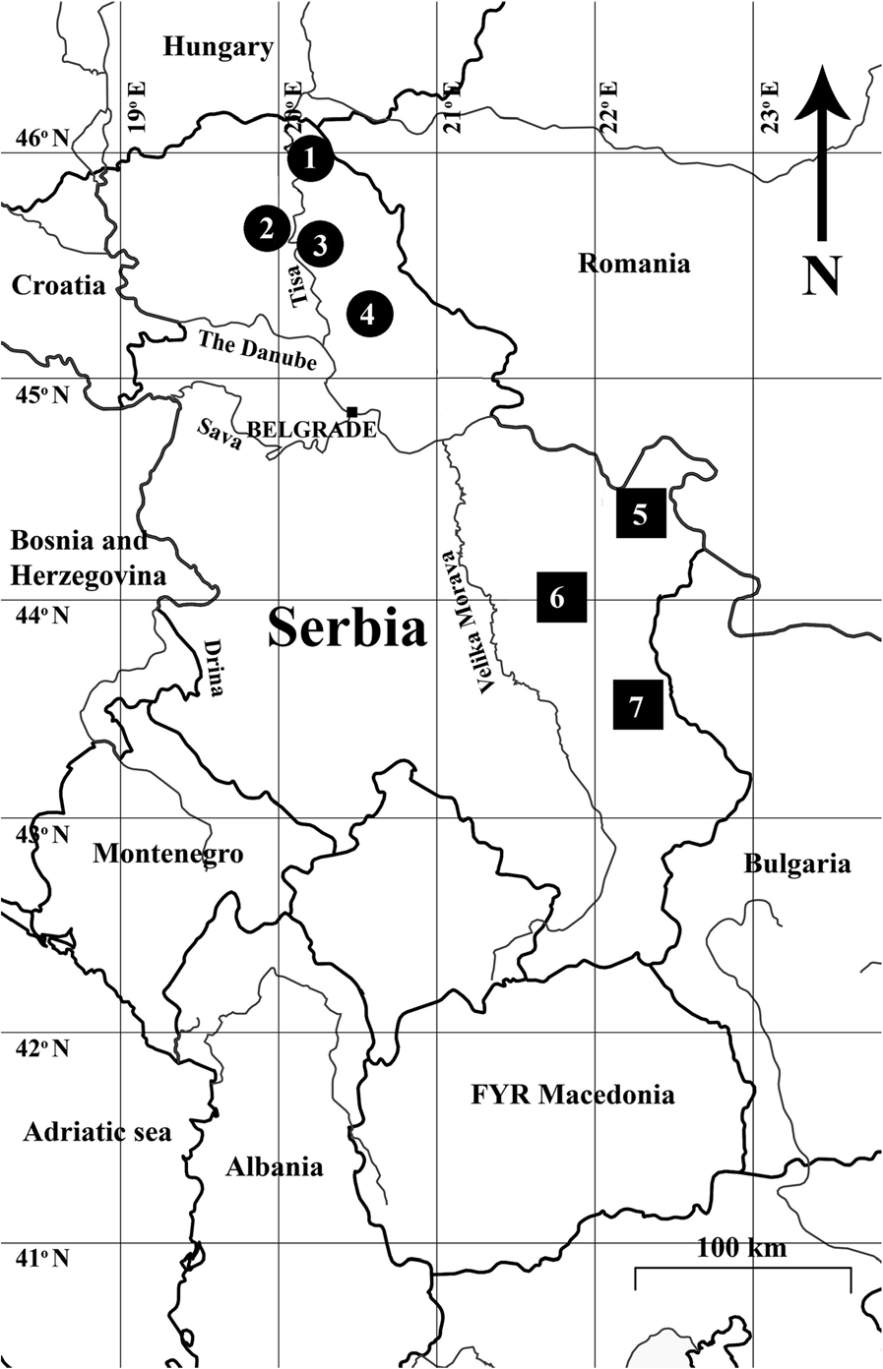
Map of Serbia with sampled localities. Circles designate populations samples from open habitats, squares from closed habitats (see Table 3 and Milošević-Zlatanović et al. [18] for full description)

Morphometric data were collected on 572 roe deer during hunting seasons from 1990. to 1995. at seven localities throughout the Republic of Serbia (Fig. 6, Table 2) along a transect spanning 400–450 km from north-east (NE) to south-west (SW). The localities and sampling have been described in detail by Milošević-Zlatanović et al. [77].

**Table 2:** Description of eleven a priori hypotheses of modularity

Hypothesis	Description of modular partitions
1	Head/Neck (1, 2, 3)
2	Trunk (4, 5, 6)
3	Forelimbs (7, 8, 9, 10)
4	Hindlimbs (11, 12, 13, 14)
5	Head/Neck+Trunk (1, 2, 3: 4, 5, 6)
6	Trunk+Forelimbs (4, 5, 6: 7, 8, 9, 10)
7	Trunk+Hindlimbs (4, 5, 6: 11, 12, 13, 14)
8	Trunk+Forelimbs+Hindlimbs (4, 5, 6: 7, 8, 9, 10: 11, 12, 13, 14)
9	Head/Neck+Trunk+Forelimbs (1, 2, 3: 4, 5, 6: 7, 8, 9, 10)
10	Head/Neck+Trunk+Hindlimbs (1, 2, 3: 4, 5, 6: 11, 12, 13, 14)
11	Forelimbs+Hindlimbs (7, 8, 9, 10: 11, 12, 13, 14)

**Fig. 7:**
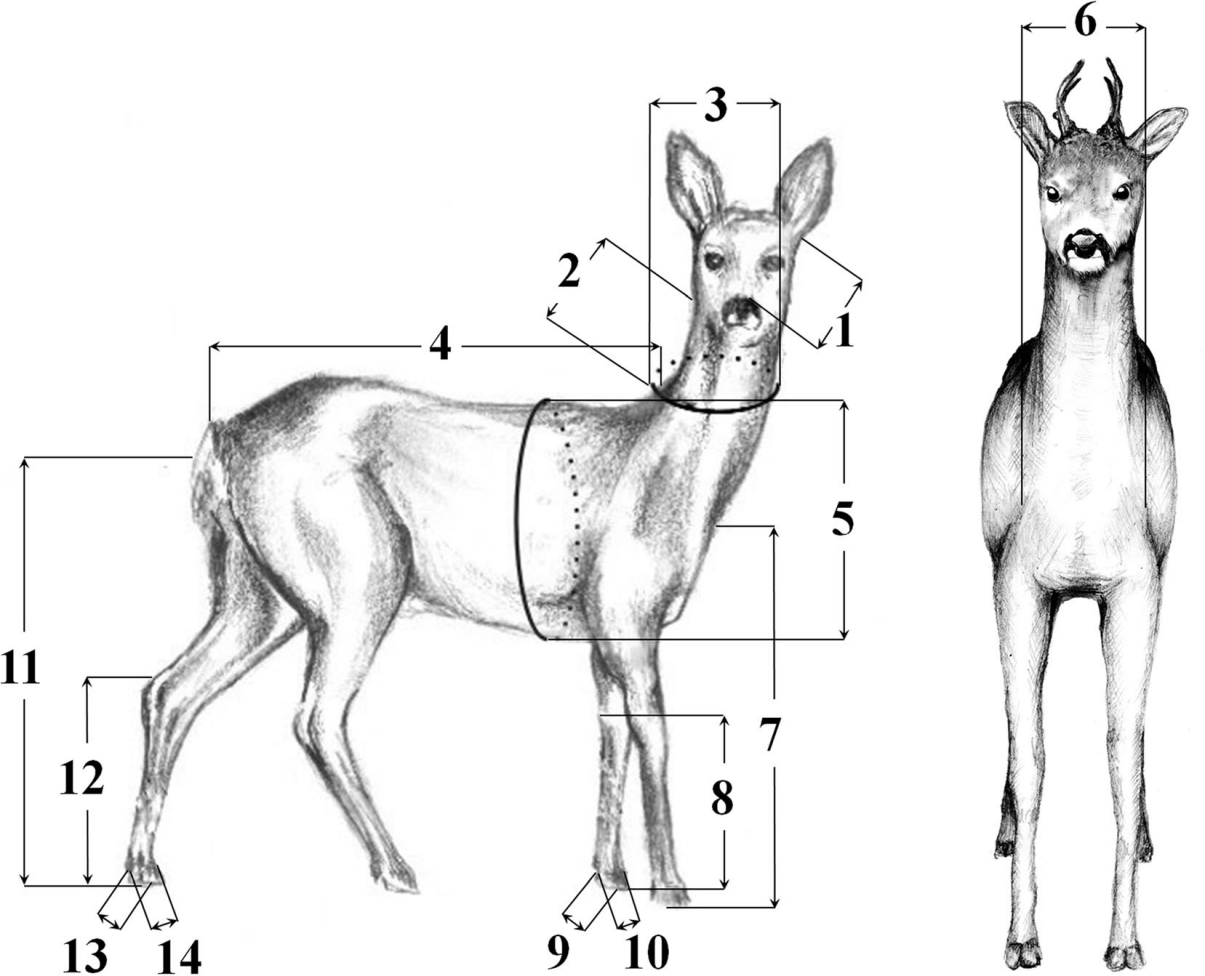
Body characters used in the analysis (lateral projection, only BD character was represented in frontal projection). The characters according to their affiliation to the analyzed modules were as follows: **HEAD/NECK**: (1) HL: Head length, Distance from the tip of the rostrum to the anterior cervical vertebrae (excluding hair); (2) NL: Neck length, Distance from the first (I) cervical vertebrae to the posterior border of the last cervical vertebrae (VII); (3) NB: Neck diameter, Distance calculated from neck perimeter; **TRUNK**: (4) TL: Trunk length, Distance from the anterior thoracic vertebrae to the posterior caudal vertebrae by following the dorsal (spinous) processes of the vertebra; (5) BB: Chest width, Distance calculated from chest perimeter; (6) BD: Chest depth, Distance of the deepest point, just behind the shoulders; **FORELIMB**: (7) FLL: Forelimb length, Distance from the tip of the hoop to the tip of *os humerus*; (8) TBL: Forefoot length or foot length of left forelimb, Distance from the end of posterior *calcaneum* to the top of hoop (or distance from the end of posterior *carpus* to the top of hoop); (9) FHL: Hoop length of left forelimb; (10) FHB: Hoop width of left forelimb; **HINDLIMB**: (11) HLL: Hindlimb length, Distance from top of hoop to the connection between *os femur* and *os ischium*; (12) HFL: Hindfoot length or foot length of left hindlimb, Distance from the end of the posterior *calcaneum* to the top of the hoop; (13) HHL: Hoop length of left hindlimb; (14) HHB: Hoop width of left hindlimb

The numbers in parentheses refer to the body characters included in each partition according to Fig. 7

Data consisted of two ontogenetic points: (1) subadult roe deer males and females (age between 14 to 24 months), and (2) adult-reproductive males and females (age > 3 years). Age was estimated by tooth wear (height of molar) [78, 79] and the weight of eye lens method [80], with subsidiary criteria being the ossification stage of the *synchondrosis spheno-occipitalis* [81], strength of pedicles (males only) and architectonics of the antlers and cranium [82]. Samples from different localities were assigned to one of the two habitat categories according to data from Milošević-Zlatanović et al. [77] based on the percentage of major habitat and foraging types: open habitats included localities with predominantly agricultural landscapes, meadows and grasslands (> 80%), closed habitats included localities situated in temperate and montane forests (> 30% continuous forest). Sample sizes of each population (locality) and habitat by sex and age, with subsamples for each sex/age/habitat groups are presented in Table 3.

**Table 3:** Population samples and habitat characteristics of the seven localities from Serbia used in the analyses

locality/population	Subadult		Adults	
	males N	females N	males N	females N
Open habitat				
1. Novi Kneževac	13	18	29	21
2. Ada-Bečej	15	14	45	81
3. Novi Bečej	21	30	62	7
4. Zrenjanin	14	10	18	30
	63	72	154	139
Closed habitat				
5. Severni Kučaj	7	6	22	25
6. Južni Kučaj	8	6	19	16
7. Stara planina	9	12	5	8
	24	24	46	49

Fourteen measurements were used to quantify body form in roe deer e.g. Milošević-Zlatanović [83] (Fig. 7). With the carcass laid flat on its side, with head and spinal column supported on the same plan, trunk length (TL) and head length (HL) were measured. With the carcass laid flat on its side and the forelimb positioned so that it was straight and perpendicular to the longitudinal axis of the body, forelimb length (FLL) and forefoot length (TBL) were measured. With the same manner the carcass and hindlimb were positioned and hindlimb length (HLL) and hindfoot length (HFL) were measured. Body measurements were recorded with a zoometric stick and measuring tape (range from 0 to 1.5 m) to the nearest 1 cm by Svetlana Milošević-Zlatanović (SMZ). The entire suite of body characters was divided into subsets reflecting the predominant shared functions, development or spatial position of body parts - basic modules: head/neck, trunk, forelimbs, hindlimbs and derived modules: head/neck+trunk, trunk+forelimbs, trunk+hindlimbs, trunk+forelimbs+hindlimbs, head/neck+trunk+forelimbs, head/neck+trunk+hindlimbs, forelimbs+hindlimbs (Fig. 2). Our driving hypothesis was that we should be able to define phenotypic modules that reflect developmental, functional and morphological aspects of the anatomy of the body and thus identify at least some modules that differ from those that were predicted purely by a priori theoretical or qualitative assumptions.

### Data adjustments and preliminary tests

Analyses of morphological integration are highly sensitive to different types of variation in the data (sexual dimorphism in size, population structuring, small sample size) [16]. As population differences are not source of variation of immediate interest for this study, the raw data were adjusted for population structuring, by adding the difference in means between the populations to the population with the smaller mean [84]. Body size and size-related variation can influence integration patterns and evolvability [60, 84, 85]. We applied the Lleonart et al. [86] normalization method to scale data and remove allometric effects. The geometric mean (GM) of 14 analysed body traits was used as an overall measure of size. For each group, we regressed each log-transformed body trait onto average GM (group-specific and log transformed). The slopes of each regression were used to adjust individual body trait following equation from Lleonart et al. [86]: Y_i_* = Y_i_[X_0_/X_i_]^b^, where Y_i_* is the theoretical value of a trait given the group average size for individual i, Y_i_ and X_i_ are the values of the specific trait and overall size (GM) for individual i, respectively, X_0_ is the group average size (average GM) and b is the coefficient of allometry for each trait [60, 84, 85]. To compare groups, allometrically adjusted data for each individual were then adjusted by its average size, scaling all individuals in all groups to a theoretical body size of 1. Allometrically adjusted data was used to derive phenotypic correlation and variance-covariance matrices, except for partial correlation analysis where we used raw data adjusted for population structuring.

A preliminary three-way (habitat/sex/age) MANOVA was performed on allometrically adjusted data to analyze the effect of these factors on body traits. The results showed significance of all three factors and their interactions, therefore further analyses were conducted on eight sex/age/habitat groups: Female/Subadult/Open (FSopen), Female/Subadult/Closed (FSclosed), Female/Adult/Open (FAopen), Female/Adult/Closed (FAclosed), Male/Subadult/Open (MSopen), Male/Subadult/Closed (MSclosed), Male/Adult/Open (MAopen), Male/Adult/Closed (MAclosed). For these eight identified sex/age/habitat groups, we additionally performed a principal coordinate analysis (PCoA) of their multivariate distance matrix according to the procedure outlined in Jamniczky and Hallgrímsson [64]. The procedure consists of computing the complement of the correlation between each pair of covariance matrices for the identified groups and subjecting the obtained 8 × 8 distance matrix to PCoA. The result of this analysis is presented graphically.

### Patterns of correlation

The Pearson correlation matrices were generated for each of eight sex/age/habitat groups using 14 allometricaly adjusted body traits. Correlation matrix repeatability was assessed with a Monte Carlo simulation (1000 replicates) to estimate the impact of sampling error. The dataset was resampled with replacement and the correlation matrices were re-estimated 1000 times [12].

Sex/age/habitat correlation matrices were subjected to matrix correlation analysis and Mantel’s test to show whether the matrices were more similar to each other or to randomly generated matrices. Significance of the matrix correlations was confirmed when the observed matrix correlation exceeded 95% of the randomly generated correlations.

### Patterns of modularity

To test integration/modularity hypotheses, the sex/age/habitat group correlation matrices were compared to connectivity matrices representing hypothesized integration of body traits. Connectivity matrices were constructed by placing a one where two traits are hypothesized to be integrated and a zero where integration was not hypothesized [12]. Matrix correlation was used to measure the correlation between the group-specific matrix and the connectivity matrices. Significance was assessed by a Mantel’s test where the observed matrix correlation is compared to an empirically derived distribution of matrix correlations 1000 times and if the observed correlation exceeds 95% of the random correlations, then the matrices are considered to be significantly similar at *p* = 0.05 [87]. We first tested for the existence of basic (a priori) modules (see Fig. 2, Table 2, first four items). This enables us to test whether the correlations among body traits of the hypothesized module are significantly higher than between all other body traits. We thus ascertain the visibility of that particular trait combination as a module since the other traits were hypothesized to be non-modularly organized. Further, after testing for visibility of basic modules, more complex matrices of modular organization were constructed by systematically combining basic modules according to developmental and functional considerations relevant to the roe deer body plan, and tested for their visibility in the same manner as for the basic modules (see Fig. 2, Table 2, items 5–11). Thus, all possible biologically relevant combinations of modularity in body traits were tested for all sex/age/habitat groups. Statistically significant integration/modularity hypotheses were additionally compared by likelihood ratio tests in order to determine which of the hypothesis better explains the data [88, 89]. A hypothesis is considered significantly better than the other if twice the difference between log-likelihood values is larger than 3.841, which is the critical value for a χ^2^ distribution with 1 d.f. and α = 0.05 [89].

### Partial correlations

Patterns of correlation were also examined using partial correlation analysis with the edge exclusion deviance statistic [90], as a significance test for conditional independence of body parts. Partial correlations measure associations between two traits that remain when underlying, shared correlations with other traits have been removed. The significance of partial correlations was assessed with the edge exclusion deviance (EED) and the χ^2^ distribution: EED = − N ln(1-ρ^2^_ij(K)_), where N is the sample size and ρ^2^_ij(K)_ is the partial correlation coefficient between variables i and j [90]. Two variables are conditionally independent when the EED value is less than 3.841 (corresponding to *p* = 0.05; d.f. = 1 from the χ^2^ distribution). For partial correlation analysis, we used raw data adjusted for population structuring following the recommendations of Magwene [90]. From the data on edge exclusion deviance, we constructed eight conditional independence graphs for each age group with two sexes and the two habitats, as described by Magwene [90]. The aim of conditional independence graphs is to visualize the patterns of phenotypic integration, to assess differences and parallelism in integration/correlation levels between age, sex and habitats. To quantify these differences, we estimated two parameters from each graph. We calculated the average connectivity and strength per trait (average number of significant edges and their strength) for each group [91]. In order to estimate differences between groups we used a General Linear Model (GLM) with connectivity and strength as dependent variables and age, sex and habitat as categorical predictors.

### Index of integration

The degree of overall correlation of body traits for each of eight sex/age/habitat groups was estimated by the index of integration, which was calculated as the variance of eigenvalues (VE) [92]. Eigenvalue variance of the correlation matrix (derived from allometrically adjusted data) was standardized by the maximum possible eigenvalue variance to allow comparison between groups [93]: VE_SD_ = VE/N-1, where VE_SD_ is the standardized eigenvalue variance, VE is the observed eigenvalue variance and N is the number of traits in the correlation matrix. Higher correlation among traits corresponds to higher values of VE and vice versa. The significance of differences in eigenvalues between groups was calculated by resampling the data with replacement and re-computing the VE [87]. The *p*-value was obtained as the number of times the VE in the group with smaller VE exceeds the bootstrapped values in the group with larger VE, divided by the number of iterations (i.e., 1000) [60].

### Evolvability indices

The ability of the body to respond to selection was evaluated via methods proposed by Hansen and Houle [94] which are derived from Lande’s [95] multivariate selection equation: Δz = Gβ, where Δz is the response vector, G is the genetic covariance matrix and β is a selection vector. We used the phenotypic covariance matrix P as a substitute for G, as several studies have shown that phenotypic and genetic covariance matrices are proportional and similarly structured [96, 97].

The covariance matrix for each of eight sex/age/habitat groups was subjected to 1000 randomly generated selection vectors and the angle between selection and response vectors was calculated. The mean cosine of the 1000 angles between selection and response vectors is the mean evolvability for each sex/age/habitat group [85]. Since mean evolvability describes the degree to which the response and selection vectors are aligned in multivariate space, the evolvability close to 1 corresponds to high respond of population mean and the evolvability close to 0 implies more constrained variation as the traits became more integrated. Statistical significance of the differences in the evolvability index was assessed by the same resampling approach as in the VE method [85]. We also computed conditional evolvability (ability of a given population to evolve in the direction of selection while under stabilizing selection) and constraints (the relative influence of PC1, which accounts for largest portion of phenotypic variance within a group, on the response to selection) as outlined by Marroig et al. [63].

The calculations were made using PopTools 2.6.2., CSIRO, Canberra [98], selection and response vectors and evolvability indices were performed with “EvolQG” [99], PCoA analysis with the cmdscale function and comparison of likelihood surfaces of integration/modularity hypotheses with “EMMLi” R package by using R 3.1.2 software [88, 100].

## Abbreviations

BB: Chest width
BD: Chest depth
FAclosed: Female/Adult/Closed
FAopen: Female/Adult/Open
FHB: Hoop width of left forelimb
FHL: Hoop length of left forelimb
FLL: Forelimb length
FSclosed: Female/Subadult/Closed
FSopen: Female/Subadult/Open
HFL: Hindfoot length or foot length of left hindlimb
HHB: Hoop width of left hindlimb
HHL: Hoop length of left hindlimb
HL: Head length
HLL: Hindlimb length
MAclosed: Male/Adult/Closed
MAopen: Male/Adult/Open
MSclosed: Male/Subadult/Closed
MSopen: Male/Subadult/Open
NB: Neck diameter
NL: Neck length
TBL: Forefoot length or foot length of left forelimb
TL: Trunk length
